# Decoding intra-tumoral spatial heterogeneity on radiological images using the Hilbert curve

**DOI:** 10.1186/s13244-021-01100-8

**Published:** 2021-10-30

**Authors:** Lu Wang, Nan Xu, Jiangdian Song

**Affiliations:** School of Health Management, China Medical University, No. 77 Puhe Rd, Shenbei District, Shenyang, 110122 Liaoning China

**Keywords:** Visual processing, Semantics, Pulmonary nodule, Solitary, Machine intelligence

## Abstract

**Background:**

Current intra-tumoral heterogeneous feature extraction in radiology is limited to the use of a single slice or the region of interest within a few context-associated slices, and the decoding of intra-tumoral spatial heterogeneity using whole tumor samples is rare. We aim to propose a mathematical model of space-filling curve-based spatial correspondence mapping to interpret intra-tumoral spatial locality and heterogeneity.

**Methods:**

A Hilbert curve-based approach was employed to decode and visualize intra-tumoral spatial heterogeneity by expanding the tumor volume to a two-dimensional (2D) matrix in voxels while preserving the spatial locality of the neighboring voxels. The proposed method was validated using three-dimensional (3D) volumes constructed from lung nodules from the LIDC-IDRI dataset, regular axial plane images, and 3D blocks.

**Results:**

Dimensionality reduction of the Hilbert volume with a single regular axial plane image showed a sparse and scattered pixel distribution on the corresponding 2D matrix. However, for 3D blocks and lung tumor inside the volume, the dimensionality reduction to the 2D matrix indicated regular and concentrated squares and rectangles. For classification into benign and malignant masses using lung nodules from the LIDC-IDRI dataset, the Inception-V4 indicated that the Hilbert matrix images improved accuracy (85.54% vs. 73.22%, *p* < 0.001) compared to the original CT images of the test dataset.

**Conclusions:**

Our study indicates that Hilbert curve-based spatial correspondence mapping is promising for decoding intra-tumoral spatial heterogeneity of partial or whole tumor samples on radiological images. This spatial-locality-preserving approach for voxel expansion enables existing radiomics and convolution neural networks to filter structured and spatially correlated high-dimensional intra-tumoral heterogeneity.

**Supplementary Information:**

The online version contains supplementary material available at 10.1186/s13244-021-01100-8.

## Key points

- The Hilbert curve may overcome the bottleneck of intra-tumor heterogeneity analyses.
- Time-consuming three-dimensional filtering of multi-scale receptive fields on images could be avoided.
- The spatial-locality–preserving approach for voxel expansion enables the filtering of high-dimensional intra-tumoral heterogeneity.

## Background

Texture plays an important role in imaging-assisted tumor detection, efficacy evaluation, and survival prognosis [1–3]. Primitive qualitative or quantitative textural descriptors for measuring tumor characteristics have been proposed using tumoral patterns that radiologists can intuitively perceive with the unaided eye [4]. With the development of data analysis and image scanning, the traditional texture descriptors may not meet the current needs of exploring latent semantics underlying high-resolution radiological images [5, 6]. Intra-tumoral heterogeneity, which reveals the co-existence of multiple subclones with distinct molecular profiles within a single tumor [7, 8], was first proven in the field of molecular image analysis by decoding deeper spatial and temporal heterogeneity. Intra-tumoral heterogeneity provides an opportunity for exploring latent semantic decoding on radiographic images [9]. Instead of capturing shallow textures through visual perception, a more nuanced method could be used, mining tumor phenotype diversity through the statistics of a distribution map of specific gray-level intensity within the partial or whole tumor region to reveal imperceptible evidence of tumor progression, recurrence, and prognosis [10–12]. Represented by the emerging field of radiomics in medical image analysis, decoding intra-tumoral heterogeneity has greatly expanded the knowledge of image phenotypic characteristics [13, 14].

However, both traditional textural descriptors and emerging latent intra-tumoral heterogeneous measurements are limited by the ability to analyze heterogeneity on only a single slice or patch of the region of interest within context-associated slices [15, 16]. Currently, decoding intra-tumoral spatial heterogeneity using the whole three-dimensional (3D) tumor sample is rare. The widely used mainstream radiomics solutions, such as Pyradiomics [14], extract thousands of delicate phenotypic features from the run-length or co-occurrence matrix from single images using advanced wavelet or Fourier transformation. Although prolific intra-tumoral heterogeneity descriptors have been reported, exploring high-dimensional intra-tumoral spatial heterogeneity is hindered by the lack of an appropriate method to describe the spatial correspondence of intra-tumoral voxels. However, decoding radiological intra-tumoral spatial heterogeneity is essential because radiological images have the distinct advantage of whole-tumor sampling, ensuring that no intra-tumor region of genetic or pathological variation is omitted [17, 18].

In current phenotypic analyses, the bottleneck in decoding intra-tumoral spatial heterogeneity on radiological images is flattening all tumor voxels into a two-dimensional (2D) matrix while maintaining between-voxel spatial structure. In such a transformation, extracting 2D intra-tumoral heterogeneity descriptors from different receptive fields on the 2D matrix is equivalent to extracting spatial intra-tumoral heterogeneity on the corresponding 3D tumor mass. Studies suggest that decoding spatial intra-tumoral heterogeneity will provide higher-dimensional image data sources, further facilitating future radiomics and artificial intelligence-based medical image analysis [19–21]. Therefore, an intuitive, feasible approach to mapping 3D (voxel) to 2D (pixel) space will overcome the current barriers to intra-tumoral spatial heterogeneity decoding.

We used a Hilbert curve-based approach to expand the tumor voxels to the 2D plane while preserving voxel spatial locality in this study. With the proposed spatial transformation, quantitative analysis of intra-tumoral spatial heterogeneity in whole tumor samples could be possible with the existing technology.

## Methods

### Hilbert curve

The Hilbert curve, *H*(*t*), or Hilbert space-filling curve, is a continuous fractal space-filling curve [22], a surjective mapping from the interval of the real number [0, 1] to the plane of the real number [0, 1] × [0, 1]. That is, given a point (*x*_0_, *y*_0_) on the plane unit square, the parameter *t*_0_ can be found using *H*(*t*) as follows:

$$H\left( {t_{0} } \right) = \left( {x_{0} , y_{0} } \right)$$

**Fig. 1:**
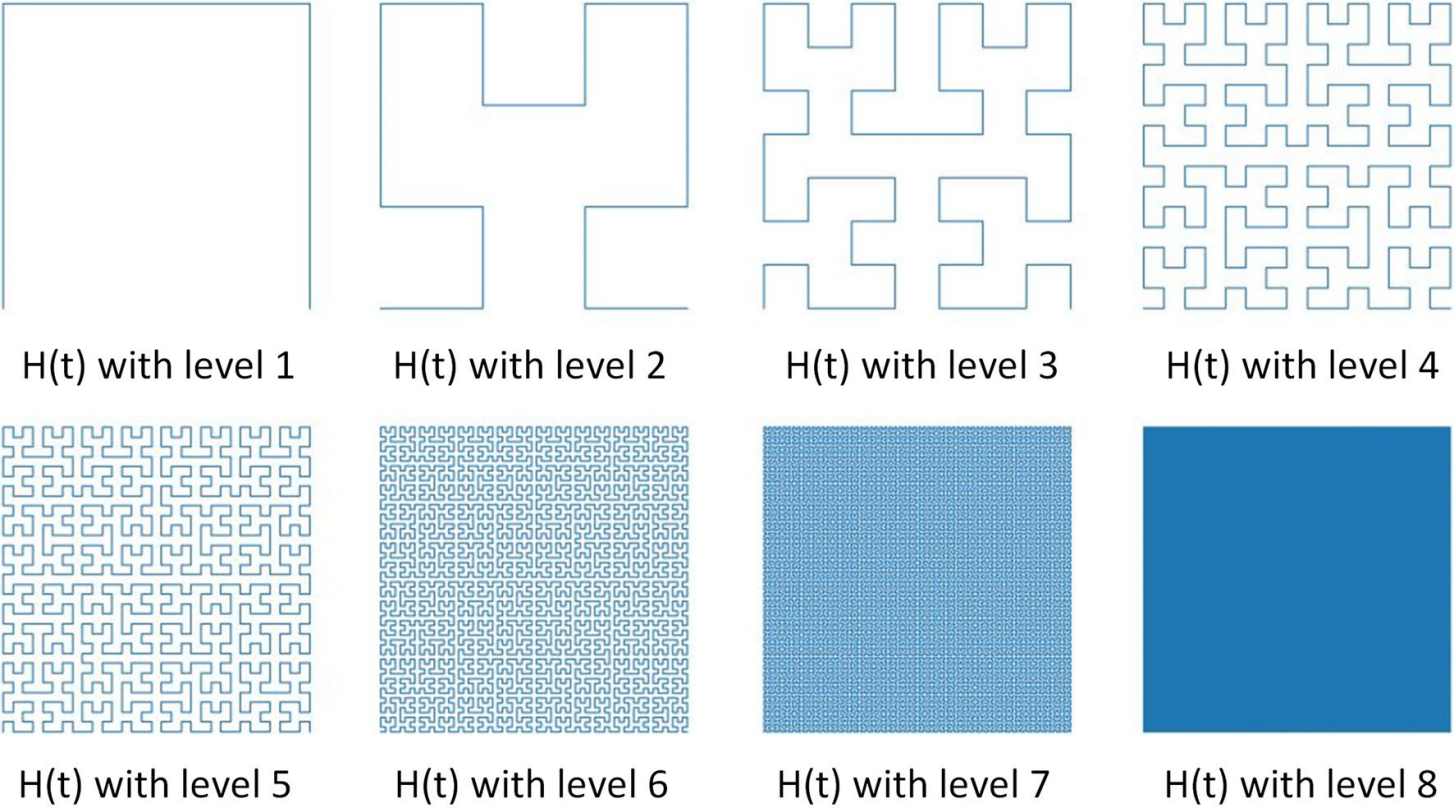
The Hilbert curve *H*(*t*) from level 1 to level 8. The size of the surjective plane unit square ranges correspondingly from $2^{1}$ × $2^{1}$ to $2^{8}$ × $2^{8}$

An illustration of the Hilbert curve with levels 1 to 8 is presented in Fig. 1.

**Fig. 2:**
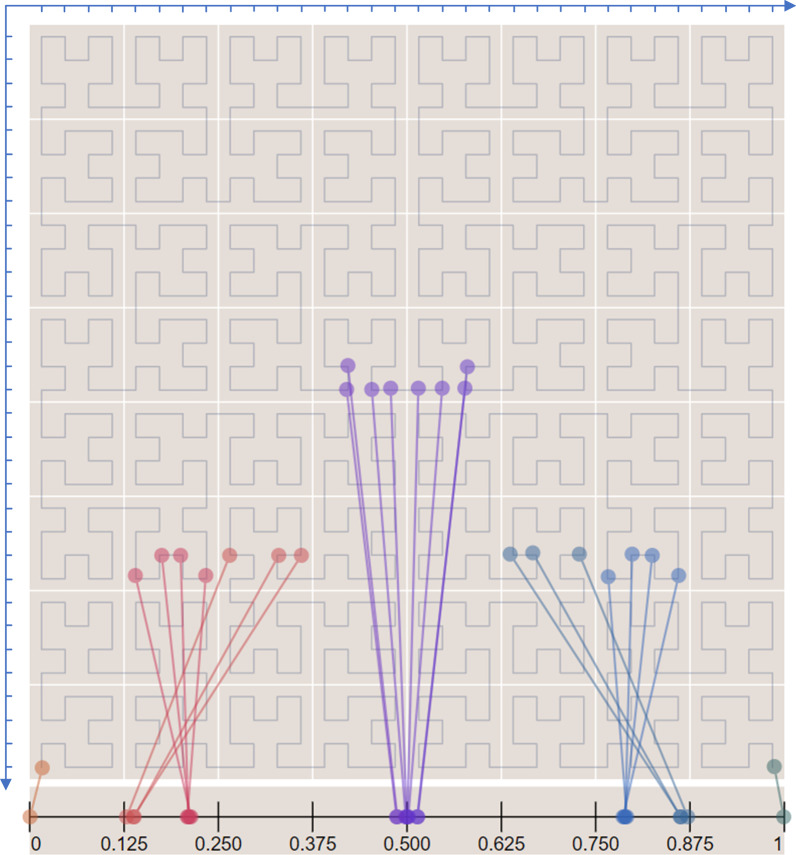
The mapping of the two-dimensional space to one-dimensional space by a Hilbert curve with level 5. The local adjacency is well preserved by mapping the points in the two-dimensional space to the one-dimensional space. The platform is obtained from http://bit-player.org

The original Hilbert curve provided a mapping between one-dimensional (1D) and 2D space, preserving the locality fairly well [23]; when traversing 2D pixels by the Hilbert curve, pixels adjacent to a certain pixel in 2D space were in close proximity to that pixel in the corresponding 1D space (Fig. 2).

Because of the locality property, the Hilbert curve effectively reduces dimensionality [24], which is the description of the information in the N-dimensional space using the (N-1)-dimensional space.

**Fig. 3:**
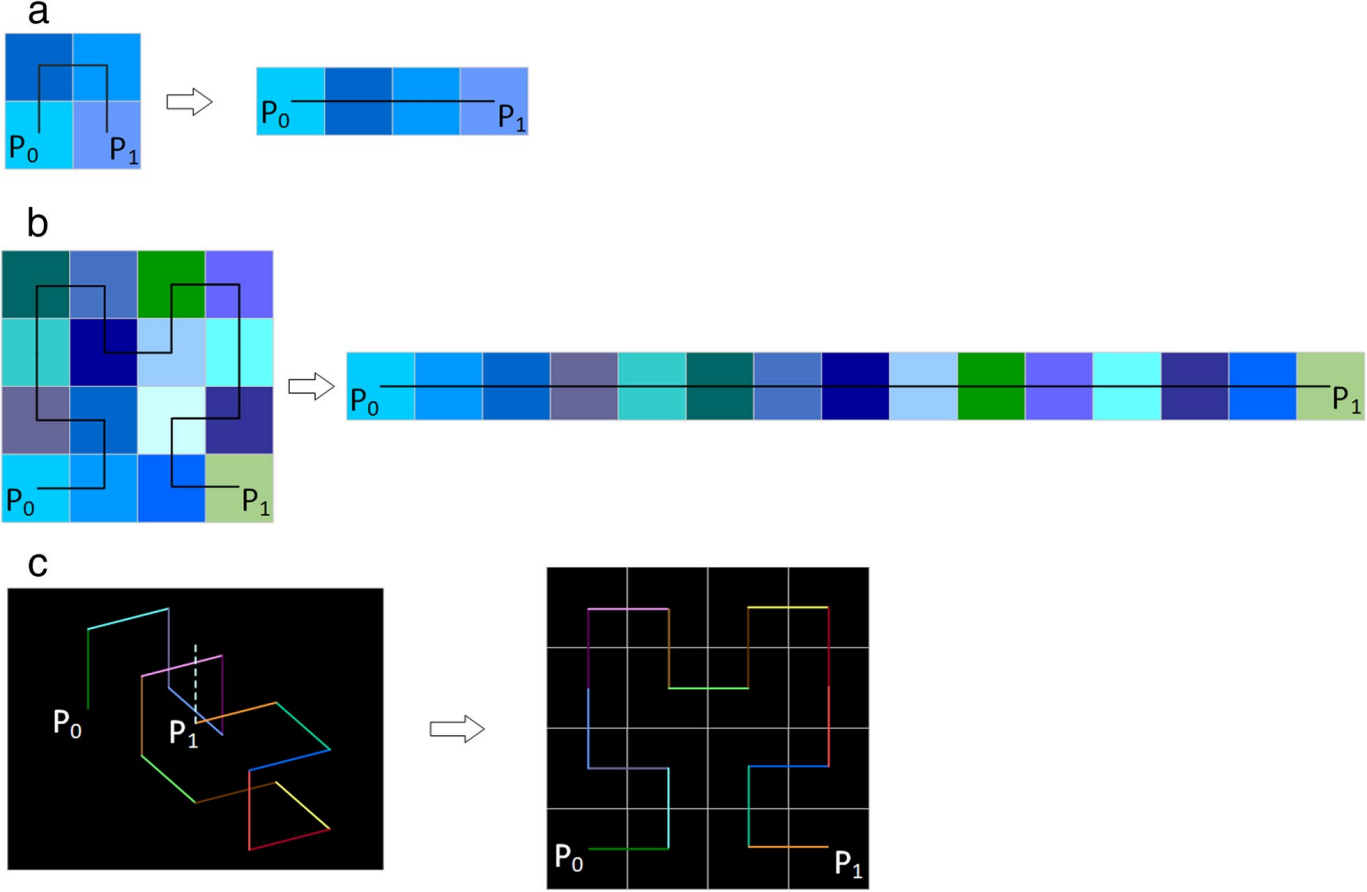
Two-dimensional Hilbert curves with level 1 (**a**) and level 2 (**b**) were, respectively, stretched into a straight line from $P_{0}$ to $P_{1}$. **c** Example of the dimensionality reduction of a three-dimensional mass with 16 voxels to a two-dimensional matrix with 4 × 4 pixels using a three-dimensional Hilbert curve

The 2D-Hilbert curve provides a mapping in which all the 2D pixels are expanded into a 1D space (which can be stretched into a straight line); each pixel’s locality with its 2D neighbors is preserved after expansion [25]. As shown in Fig. 3a, an image with four pixels from *P*_0_ to *P*_1_ could be traversed by a level 1 Hilbert curve by expanding the image with a width of two and a height of two to a 1D space with four points. The level 2 Hilbert curve enables filling a 16-pixel image, transforming a 2D image with a width of four and a height of four to a 1D space with 16 points (Fig. 3b). As the Hilbert curve iteration increases, the size of the image that can be filled increases correspondingly. As the iteration approaches infinity (that is, a 2D plane with an infinite number of pixels), the space will be filled by the Hilbert curve.

Further, an object in 3D space, expressed by a 3D Hilbert curve, could be expanded to 2D space, and the neighboring properties of spatially adjacent voxels would be maintained on the 2D image (Fig. 3c).

Therefore, in image analysis, with the help of a 3D Hilbert curve, current intra-tumoral heterogeneity analysis techniques could be employed on 2D images to interpret information inherent in the 3D volume by characterizing the spatial correlation into 2D images. When using a 3D Hilbert curve for dimensionality reduction, local voxel adjacency in 3D space is well preserved on the corresponding 2D image after Hilbert expansion, as shown in Fig. 3c.

### Dimensionality reduction for voxel expansion

**Fig. 4:**
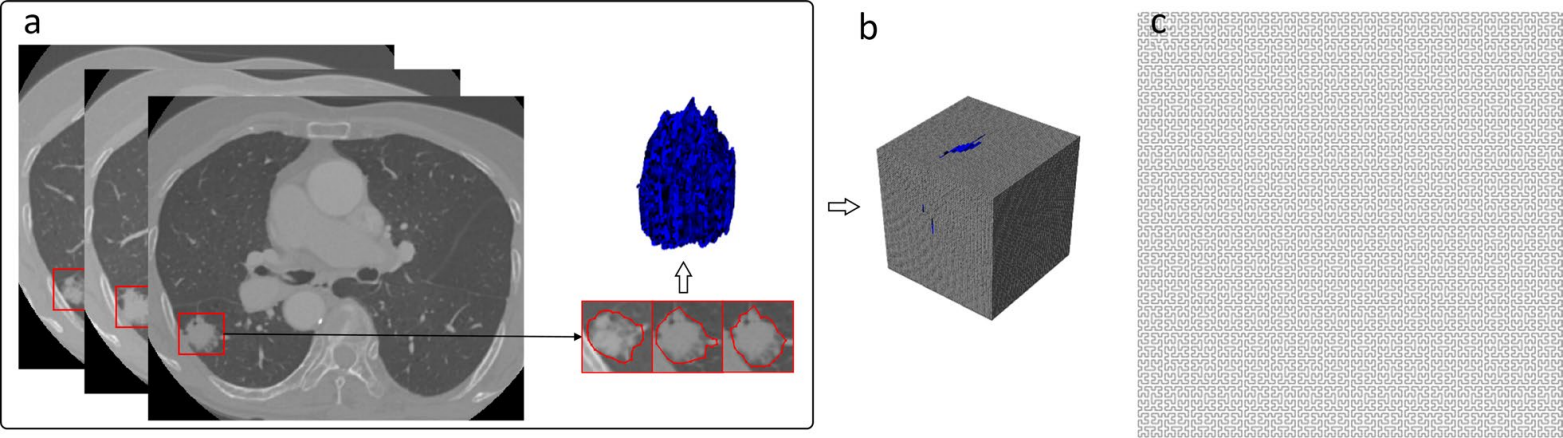
**a** The lung tumor used to illustrate the three-dimensional (3D) Hilbert curve expansion. **b** The Hilbert volume with level 6 defined in this study, and the lung tumor (in blue) encapsulated in the Hilbert volume according to the 3D coordinates. **c** The empty Hilbert matrix to store the result of dimensionality reduction from (**b**)

CT scans of patients from the open-access Lung Image Database Consortium image collection (LIDC-IDRI) database [26] were used in this study for dimensionality reduction. Manual segmentation of the lung tumor was performed by one to four radiologists, and the intersection of the radiologists was used. One of the CT scans including a lung tumor spreading across 43,068 voxels in 31 slices in total, was used to illustrate the procedure. Informed consent was not required for the data, and the lung tumor is presented in Fig. 4a.

In this study, a level 6 3D Hilbert curve was defined to store the lung tumor voxels, which we called the Hilbert volume, to reduce the 3D tumor volume dimensionality to a 2D matrix. The size of the Hilbert volume was $\left( {2^{6} , 2^{6} , 2^{6} } \right)$. We rescaled the tumor’s maximum diameter to $< 2^{6}$ on the axial, sagittal, and coronal planes, respectively. The real gray intensity of the tumor voxels on CT was used in the Hilbert volume defined, and the others were marked as zero.

Next, a 2D Hilbert curve with the same number of points as the 3D Hilbert volume (Fig. 4b) was defined to store all the pixels transformed from the 3D Hilbert volume voxels, which we called the Hilbert matrix. The size of the Hilbert matrix was $2^{9} \times 2^{9}$ as shown in Fig. 4c.

Then, we simulated reducing the 3D space to 2D space. During dimensionality reduction, each image layer on the cross-sectional axis was sequentially input into the Hilbert transformation. Slice layers pulled from the 3D Hilbert volume were pushed to the corresponding positions on the 2D Hilbert matrix where the previous slice was located to demonstrate the dimensionality reduction vividly; the points of the previous slice were moved outward step-wise along the continuous fractal space-filling curve. This process was iterated until all image layers were pushed to the Hilbert matrix. Finally, the Hilbert matrix was filled in, and all voxels fell on the corresponding pixel position, as defined by the Hilbert curve.

**Fig. 5:**
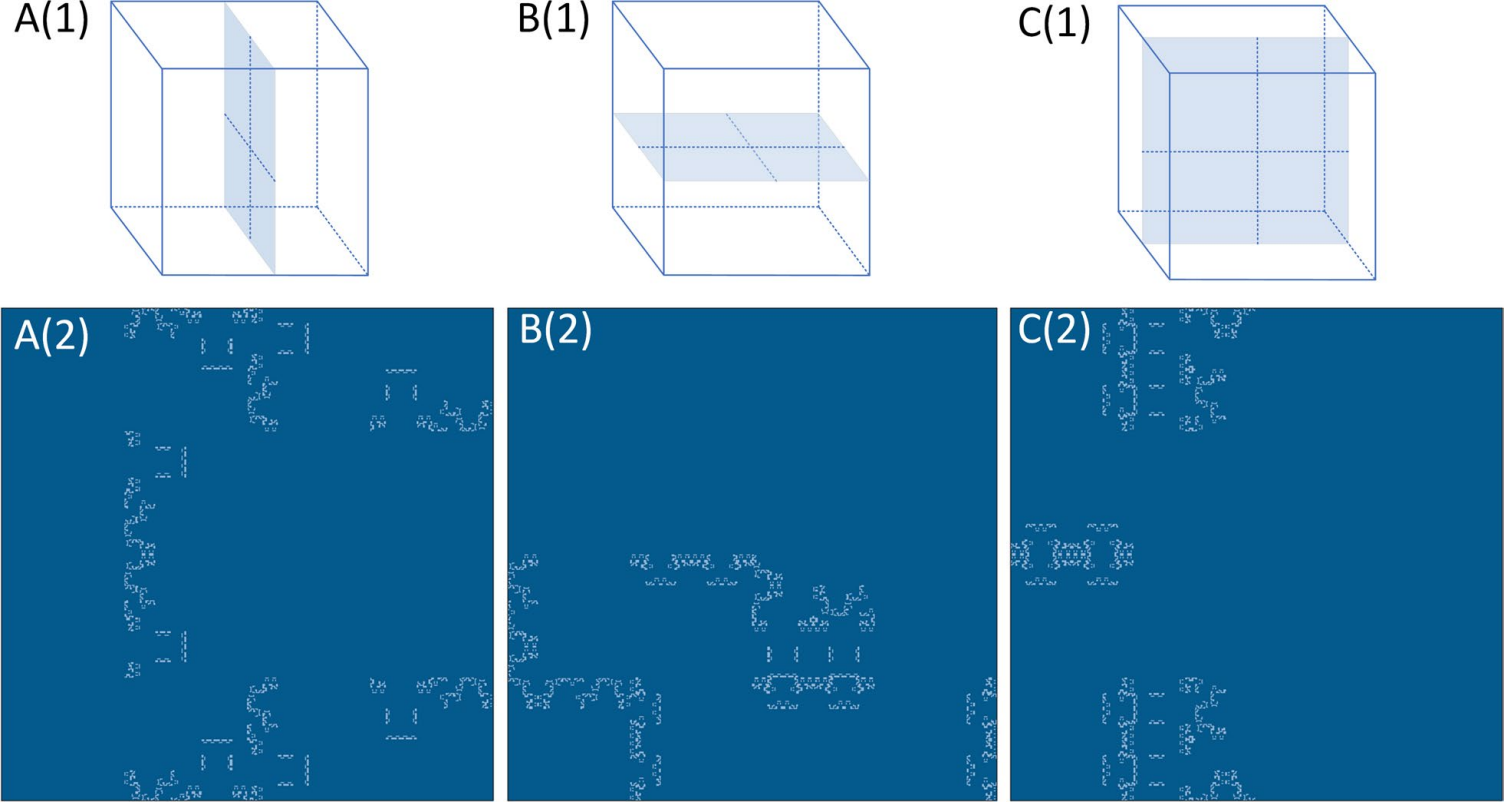
The Hilbert volumes containing only a single image (the first row). The corresponding expansion to a Hilbert matrix demonstrated by the proposed Hilbert curve-based mapping approach (the second row)

**Fig. 6:**
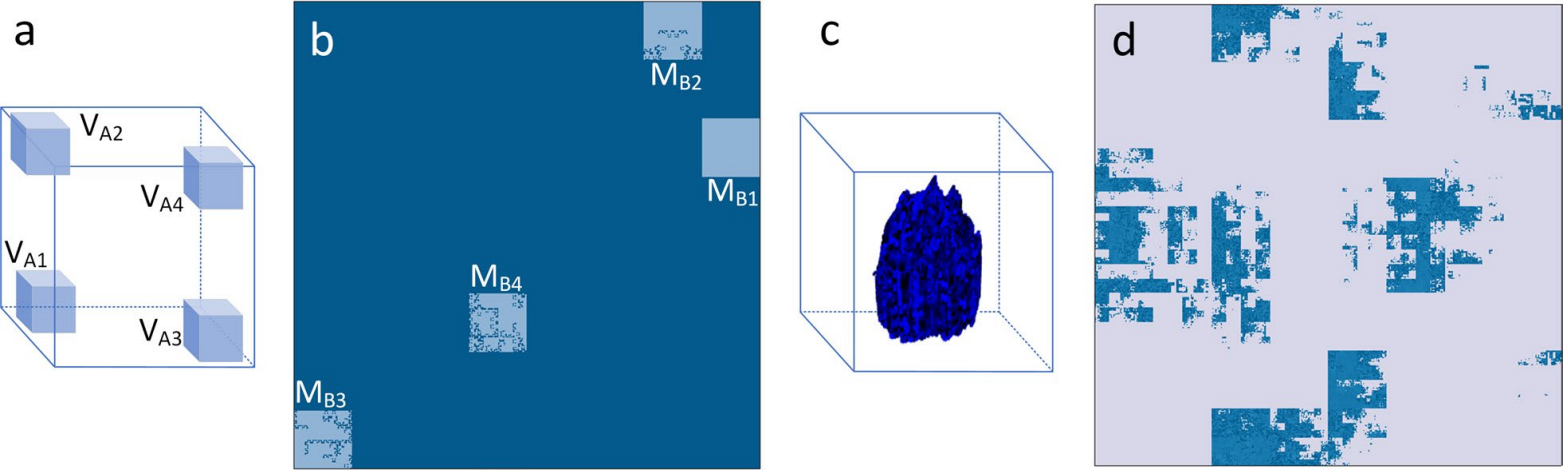
**a** A Hilbert volume consisting of four blocks of V_A1_, V_A2_, V_A3_, and V_A4_ was expanded into a Hilbert matrix (**b**). The corresponding expansions of the four blocks were M_B1_, M_B2_, M_B3_, and M_B4_ on the matrix, respectively. **c** The Hilbert volume with the lung tumor inside was expanded to a two-dimensional Hilbert matrix (**d**). Accordingly, the voxels of the lung tumor (**c**) in blue were expanded into the pixels of the matrix (**d**) in blue

To demonstrate the spatial locality after the Hilbert curve-based voxel expansion, the following experiments were performed:

1. Three Hilbert volumes with level 6 were constructed, consisting of only a single image on the axial, coronal, and sagittal planes; other voxels were marked as zero, as shown in Fig. 5a. The Hilbert volumes, containing only one image on the traditional planes, were used to illustrate the expansion results of the proposed Hilbert curve-based spatial correspondence mapping approach for the single slice on the traditional planes. All three Hilbert volumes were then expanded to the corresponding Hilbert matrices.
2. A Hilbert volume with level 6 consisting of four 3D blocks with sizes of $\left( {2^{4} , 2^{4} , 2^{4} } \right)$ was constructed; other voxels were marked as zero, as shown in Fig. 6a. The Hilbert volume constructed here was used to clarify the difference of the expansion results by the proposed approach between the 3D blocks and the slices. The location of the blocks varied inside the volume.
3. A Hilbert volume with level 6 with a lung tumor inside was expanded into the Hilbert matrix, as shown in Fig. 6c. The Hilbert volume constructed with the lung tumor was used to indicate the intra-tumor spatial heterogeneity decoded by the proposed Hilbert curve-based spatial correspondence mapping approach.
4. According to the latest LIDC-IDRI nodule list released [27], all the 2635 lung nodules, including 14,266 CT images, were used to evaluate the performance of the Hilbert matrix image on the task of classification into benign or malignant masses. The LIDC-IDRI dataset was used because all the nodules were diagnosed by at least one radiologist, and scoring as malignant or benign was provided by the radiologists. In addition, all the lung nodules were manually delineated by at least one radiologist. The averaged malignancy rating for each nodule and the intersection of the segmentation of each nodule from the radiologists were used in this study. As described by a previous study [28], nodules with an average score < 3 were classified as benign; those with an average score > 3 as malignant. A state-of-the-art network for classification, named Inception-V4 [29], was used to test both the Hilbert matrix images of lung nodules and the original lung nodule CT images. Lung nodules with a minimum diameter of 5 mm were used and divided into training, validation, and test datasets (80%:10%:10%); the difference of classification accuracy was evaluated by the McNemar's test. Furthermore, to validate the robustness of the proposed approach, we used the manual nodule segmentation in the test dataset only performed by Radiologist 1 of the LIDC-IDRI dataset. The Hilbert curve-based spatial correspondence mapping was implemented to the segmented nodules, and the Hilbert matrix images were obtained. All the images were then input into the well-trained Inception-V4 model to verify the potential bias caused by segmentation.

The source code for Hilbert curve-based spatial correspondence mapping in this study is publicly available at https://github.com/JD910/HilbCurv_Spatial_Heterogeneity. Hilbert volume data for this study, consisting of axial plane images, 3D blocks, and the illustrated lung tumor, are also available at the above repository to facilitate the reproduction of our results. Appropriate institutional review board approval was obtained for this study.

## Results

The voxels on the Hilbert volume and the corresponding pixels in the Hilbert matrix after applying the proposed spatial correspondence mapping are presented in Additional file 1: Appendix Video 1. The colors of the points on the curves indicate the spatial correspondence. The 3D Hilbert volume with level 4 and the corresponding 2D Hilbert matrix with level 6 were used to illustrate the details of pixel adjacency clearly.

For a better presentation of dimensionality reduction, the procedure is presented as an animation in Additional file 2: Appendix Video 2 to demonstrate the transformation of the 3D Hilbert volume of the lung tumor to the 2D Hilbert matrix. The image slices were flattened into the matrix one by one, and the pixels moved outward step by step to the corresponding position as defined by the Hilbert fractal curve. The Hilbert matrix was finally filled, and the dimensionality reduction was completed. The tumor within the Hilbert volume was preserved after dimensionality reduction in the animation to visualize better the contrast between the mass in the 3D space and the corresponding pixels in the 2D matrix.

Results of the 2D transformation of the Hilbert volumes consisting of a single image on the axial, coronal, and sagittal planes are presented in Fig. 5. The corresponding results of the Hilbert volumes consisting of 3D blocks and the lung tumor sample are presented in Fig. 6.

The results of the above experiments indicated that when transforming images that are commonly visualized in 2D space, such as the slices arranged on axial, coronal, and sagittal planes, the pixel distribution on the corresponding Hilbert matrix is scattered and irregular, difficult to analyze by radiomics or convolutional neural networks. Therefore, further analysis of the Hilbert matrix derived from such 2D slices tends to be meaningless, as shown in the second row in Fig. 5. However, for the 3D blocks inside the Hilbert volume, the result of dimensionality reduction to the 2D matrix indicated regular and concentrated squares. This finding was further demonstrated by the Hilbert matrix, which was expanded from the volume containing the lung tumor. As shown in Fig. 6d, although there were outliers in the image because of the spatial irregularity of lung tumors, almost all the voxels were expanded systematically, corresponding to the rectangular and strip-shaped areas. Applying filtering of different receptive fields to these uniform areas on the 2D matrix to detect textures and latent semantics is equivalent to filtering the corresponding 3D blocks of the same size in 3D space. Therefore, the proposed Hilbert curve-based spatial mapping to expand the tumor sample to a 2D matrix in voxels will enable radiomics and neural networks to filter more structured and spatially correlated high-dimensional intra-nodular heterogeneity using the existing techniques. A detailed explanation of how to apply the proposed method to radiomics and convolution neural networks is presented in additional file.

Lung nodules from the LIDC-IDRI dataset were used to further demonstrate the performance of the Hilbert curve-based spatial correspondence mapping approach. A total of 532 benign and 401 malignant nodules from the dataset were included, and data augment was used to balance training and test data. Detailed statistics of the samples, volume, and accuracy (with 95% confidence intervals) of the classification using the two types of images on the training, validation, and test datasets are presented in Table 1. The classification into benign and malignant nodules indicated that an accuracy of 93.45%, 86.36%, and 85.54% was obtained in the training, validation, and test datasets, respectively, when using the Hilbert matrix images decoded from the nodule volumes by the proposed Hilbert curve-based mapping method. When using the original CT images, the Inception-V4 network indicated that an accuracy of 95.69%, 75.05%, and 73.22% was obtained on the corresponding datasets. A significant difference in accuracy was found on the test dataset using McNemar’s test (*p* < 0.001). In addition, classification using the Hilbert matrix images transformed from the manual segmentation by Radiologist 1 in the LIDC-IDRI dataset showed an accuracy of 86.11% with 95% confidence interval of 79.05% to 93.17% on the test dataset.

**Table 1:** Statistics of samples, volume (presented by the LIDC-IDRI dataset), and accuracy (with 95% confidence intervals) of classification using the two types of images on the training, validation, and test datasets

	Training	Validation	Test
Dataset			
Benign	428	52	52
Malignant	321	40	40
Volume (avg)	1221.05	985.33	1069.61
Accuracy (95% CI)			
Original CT images	95.69% (94.29–97.09%)	75.05% (66.21–83.89%)	73.22% (64.18–82.26%)
Hilbert matrix images	93.45% (91.86–95.04%)	86.36% (69.35–93.37%)	85.54% (78.35–92.73%)

*CI* confidence intervals

## Discussion

We explored and validated a new approach for mapping and visualizing high-dimensional tumors on radiological images into the two-dimensional space while preserving the between-voxel spatial locality. We demonstrated that the Hilbert curve is a reliable method for decoding intra-tumoral spatial heterogeneity to overcome the bottleneck of current intra-tumor heterogeneity analyses, which can only be performed on single slices or regions of interest within context-associated radiological slices. Our experiments with traditional axial plane images, three-dimensional blocks, and lung tumor samples demonstrated the superiority of the proposed method for preserving spatial locality when expanding the entire three-dimensional tumor sample into a two-dimensional matrix. By designing a specific volume-oriented receptive field of filters on the matrix, this approach holds promise for intra-tumoral spatial heterogeneity extraction of whole or partial tumors of various sizes in the corresponding two-dimensional space using current mature radiomics or deep learning techniques.

Extracting intra-tumoral spatial heterogeneity from 3D tumor samples on radiological images is challenging because the current technique is limited to analyzing conventional 2D image slices [13, 30]. Radiomics-based heterogeneity analysis studies primarily use features extracted from the run-length matrix, co-occurrence matrix, and wavelet derived from a single slice [31]. This scheme has been accepted as the mainstream workflow for radiomics-based toolkit development, such as Pyradiomics [14]. Although studies have proposed using the average of several internal layers of the tumor to calculate the intra-tumoral heterogeneity descriptors, the essence of these methods is still 2D processing. In radiomics, designing spatial intra-tumoral heterogeneous descriptors from the perspective of 3D tumor samples or masses is rare.

To filter the emerging convolution neural network, a 3D kernel is applied to identify voxels covered by the kernel for feature representation. Generally, three context-associated slices are input into the kernel simultaneously; with step-by-step kernel movement, feature maps with latent spatial semantics are extracted by the convolutional neural network [32]. Although 3D convolution achieves the analysis of spatial heterogeneity, to a certain extent, it is limited by the “black box” of the filter; it is challenging to extend the filter to include more slices because increasing the 3D filter volume means an exponential surge in computational consumption [33]. Therefore, simultaneous quantitative heterogeneity analysis of tumor blocks of various sizes is difficult using this method.

We proposed a Hilbert curve-based approach to overcome these barriers, mapping the whole tumor sample to a 2D matrix while preserving the voxels’ spatial locality. The Hilbert curves and other fractal-based methods to reduce dimensionality have been explored in other domains [34, 35], and our study demonstrated that the 3D tumor blocks with sizes of $\left( {2^{4} , 2^{4} , 2^{4} } \right)$ at different locations inside the Hilbert volume were expanded to the corresponding square on the 2D Hilbert matrix. Thus, the idea of radiomics-based intra-tumoral spatial heterogeneity extraction performed on tumor samples has been transformed to the conventional heterogeneity extraction workflow on the corresponding square on the 2D matrix/image. With the help of the Hilbert curve-based dimensionality reduction proposed here, intra-tumoral spatial heterogeneity extraction of tumor samples is feasible using the Hilbert matrix-based run-length, co-occurrence, and wavelet-based feature extraction. Therefore, our method is promising for future radiomics studies to develop automatic and productive intra-tumoral spatial heterogeneity feature extraction from radiological images.

Furthermore, three or five image slices are commonly included in 3D convolution kernels [36]. The 3D kernel’s size cannot be expanded arbitrarily because the capacity and time required increase significantly as the size of the convolution kernels increases. For feature presentation of blocks inside the tumor, 2D convolution is more efficient than 3D convolution, but it sacrifices the spatial correlation within the image context. Therefore, balancing the computational efficiency and spatial context for convolutional neural networks is challenging [33]. With the proposed spatial correspondence mapping, conventional convolution with multiple receptive fields performed on the 2D matrix is equivalent to 3D convolution for the corresponding 3D blocks. Although the obtained 2D matrix is not as concrete as the common cross-sectional images are, it truly and vividly reflects the intra-tumoral spatial heterogeneity distribution, as demonstrated by the experiment on the 933 lung nodules from the LIDC-IDRI dataset. Therefore, 2D convolution to the Hilbert matrix is promising to improve the efficiency of high-dimensional convolution and maintain between-voxel spatial attributes.

Our study has several limitations. First, we only validated Hilbert curve-based spatial mapping; however, there are other space-filling curves, such as the Peano curve, Gosper curve, and Koch snowflake [37–39]. In the future, we will explore these space-filling curves’ ability to preserve the spatial locality and explore the specific correspondence between 3 and 2D space to determine the mathematical mechanism of space-filling curves in spatial intra-tumoral heterogeneity analysis. Additionally, this preliminary study only used lung tumors to illustrate and demonstrate the Hilbert curve-based tumor sample expansion. Multiple tumor samples from radiology, histopathology, and genomics should be used to explore and validate the intra-tumoral spatial heterogeneity decoding in the future. Finally, the result of unfolding the spatial heterogeneity between the intra-tumoral voxels proposed in this study is an abstract mapping of the spatial correspondence and not the conventional axial plane images. Therefore, the visual interpretation of the image needs to be explored to broaden our knowledge of the characteristics of the decoded intra-tumoral spatial heterogeneity.

In conclusion, we proposed and validated a Hilbert curve-based approach to map and visualize high-dimensional tumors from radiological images into two-dimensional images while preserving the between-voxel spatial locality. This method could overcome the bottleneck of current tumor sample-based intra-tumoral spatial heterogeneity extraction and holds promise for launching high-dimensional intra-tumoral spatial heterogeneity analyses of radiological images in radiomics and promoting neural networks to identify more structured and spatially correlated high-dimensional heterogeneous semantics.

## Supplementary Information

**Additional file 1**.** Appendix Video 1**: A demonstration of a Hilbert curve-based mapping to realize two-dimensional expansion from a three-dimensional lung nodule (in blue) volume.

**Additional file 2**.** Appendix Video 2**: Example of using a Hilbert curve-based mapping to expand a three-dimensional Hilbert volume to a two-dimensional Hilbert matrix.

## Data Availability

All the data can be downloaded from the LIDC-IDRI dataset from https://wiki.cancerimagingarchive.net/display/Public/LIDC-IDRI. All the source code and data to reproduce our results are publicly available at: https://github.com/JD910/HilbCurv_Spatial_Heterogeneity

## Abbreviations

1D: One-dimensional
2D: Two-dimensional
3D: Three-dimensional
LIDC-IDRI: Lung Image Database Consortium image collection
